# Lung Involvement in Patients with Leptospirosis in Tropical Australia; Associations, Clinical Course and Implications for Management

**DOI:** 10.3390/tropicalmed10120333

**Published:** 2025-11-26

**Authors:** Adam Sykes, Simon Smith, Hayley Stratton, Megan Staples, Patrick Rosengren, Anna Brischetto, Stephen Vincent, Josh Hanson

**Affiliations:** 1 Department of Medicine, Cairns Hospital, Cairns, QLD 4870, Australia; adam.sykes@health.qld.gov.au (A.S.); simon.smith2@health.qld.gov.au (S.S.); hayley.stratton@nt.gov.au (H.S.);; 2 Leptospirosis Reference Laboratory, Coronial and Public Health Sciences, Brisbane, QLD 4108, Australia; megan.staples@health.qld.gov.au (M.S.); anna.brischetto@health.qld.gov.au (A.B.); 3 School of Medicine and Dentistry, James Cook University, Cairns, QLD 4870, Australia; patrick.rosengren@my.jcu.edu.au; 4 The Kirby Institute, University of New South Wales, Sydney, NSW 2052, Australia

**Keywords:** leptospirosis, lung disease, pulmonary haemorrhage, clinical management, critical care, tropical medicine, pathophysiology

## Abstract

Lung involvement in patients with leptospirosis is associated with a more complicated disease course. However, the demographic and clinical associations of lung involvement are incompletely defined, and its optimal management is uncertain. This retrospective study examined consecutive patients admitted to a referral hospital in tropical Australia, with laboratory-confirmed leptospirosis between January 2015, and June 2024. Lung involvement was defined as new lung parenchymal changes on chest imaging at any point during the patients’ hospitalisation. The demographics, clinical findings and clinical course of the patients with and without lung involvement were compared. The median (interquartile range (IQR)) age of the 109 patients was 39 (24–56) years; 93/109 (85%) were male. Lung involvement was present in 62/109 (57%), 55 (89%) of whom had no documented comorbidities. Patients with lung involvement received antibiotics later in their disease course than those without lung involvement (after a median (IQR) of 5 (4–6) versus 3 (2–5) days of symptoms, *p* = 0.001). Lung involvement was frequently associated with multi-organ failure: patients with lung involvement were more likely to require intensive care unit admission than patients without lung involvement (41/62 (66%) versus 15/47 (32%), *p* < 0.001). Overall, 30/109 (28%) satisfied criteria for acute respiratory distress syndrome (ARDS) and 26/109 (24%) developed pulmonary haemorrhage. Patients with lung involvement received cautious fluid resuscitation, vasopressor support and prompt initiation of additional supportive care—including mechanical ventilation, renal replacement therapy and extracorporeal membranous oxygenation—guided by the patients’ physiological parameters and clinical trajectory. All 109 patients in the cohort were alive 90 days after discharge. Life-threatening lung involvement was identified in the majority of individuals in this cohort and occurred in young and otherwise well individuals. However, in Australia’s well-resourced health system excellent outcomes can be achieved using a standard contemporary approach to the management of a patient with undifferentiated infection while a confirmed diagnosis of leptospirosis is awaited.

## 1. Introduction

Leptospirosis is a potentially life-threatening zoonotic disease caused by pathogenic spirochetes of the genus *Leptospira*. In 2015, it was estimated that globally there were over one million human cases annually, resulting in almost 59,000 deaths [1]. The case-fatality rate of patients with severe leptospirosis who are admitted to the intensive care unit (ICU) can exceed 50% [2]. Despite this, leptospirosis remains an under recognised disease and its pathophysiology and optimal management are still incompletely understood [3,4,5,6,7].

Symptomatic leptospirosis typically presents with fever, rigors, myalgia, nausea, vomiting, and headache. In most individuals it is a mild illness, but approximately 10% of cases develop severe disease characterised by rapidly evolving multiorgan dysfunction with manifestations that can include hypotension, acute kidney injury (AKI), pulmonary haemorrhage and acute respiratory distress syndrome (ARDS) [5]. Lung involvement is often a harbinger of a more complicated disease course with death almost 10 times more common in patients with lung involvement in some series [8,9,10,11].

However, there is uncertainty about the risk factors for lung involvement and how best to manage the patients who develop this complication. Although antibiotics are recommended in patients with symptomatic leptospirosis, the incremental value of antibiotic therapy above optimal supportive care is uncertain [5,12]. Conservative fluid management strategies are frequently employed in individuals with lung involvement although achieving an optimal fluid balance is challenging in individuals who are also often hypotensive and/or have AKI [13,14,15,16]. Some observational studies have suggested a benefit of corticosteroid therapy in individuals with lung involvement; however, evidence of benefit in randomised trials is lacking [17,18]. Plasma exchange, plasmapheresis, and extracorporeal membrane oxygenation (ECMO) have been used by clinicians to treat severe leptospirosis, particularly when accompanied by pulmonary haemorrhage; however, the benefits of these interventions have only been reported in small case series [5,19,20,21]. The role of tranexamic acid and other adjunctive therapies for individuals with pulmonary haemorrhage is also incompletely defined [22].

Far North Queensland (FNQ), in tropical Australia, has the highest incidence of leptospirosis in the country; in some years the local incidence of confirmed disease can approach 15/100,000 population [23,24]. Prompt access to sophisticated multidisciplinary care in the well-resourced Australian health system means that deaths from leptospirosis are very rare in the region, even in patients with multi-organ failure requiring ICU care [14,25]. FNQ clinicians recognise that individuals with leptospirosis that is complicated by lung involvement are at greatest risk of death, but what are the characteristics of these patients and what is their optimal management? This study, performed in a high-volume centre where leptospirosis has a very low case-fatality rate, examined the demographic, clinical, laboratory and imaging findings of individuals with laboratory confirmed leptospirosis who did and did not have lung involvement. It was hoped that these data, might inform the management strategies of clinicians, less familiar with leptospirosis, who encounter individuals with this potentially life-threatening disease.

## 2. Materials and Methods

This retrospective cohort study was conducted at Cairns Hospital, the 770-bed referral hospital for the FNQ region. The hospital serves a population of approximately 290,000 who live across an area of approximately 380,000 km^2^ (Figure S1). FNQ has a hot, tropical climate; most local cases of leptospirosis occur in the December–April wet season when the average monthly rainfall can exceed 400 mm [26,27]. In approximately 85% of cases, there is a history of potential occupational or recreational exposure to the organism, with almost 90% of the cases occurring in districts where there is high-intensity banana, sugar and cattle farming [24].

This is a substudy of a previously published report which examined consecutive adults and children admitted to Cairns Hospital with a confirmed diagnosis of leptospirosis between 1 January 2015, and 30 June 2024 [25]. This study period was chosen as it coincided with the local introduction of an electronic medical record (EMR). Individuals were eligible for inclusion in the study if they satisfied the Australian definition of laboratory confirmed leptospirosis (Table S1) [28]. Culture of *Leptospira* from whole-blood, with subsequent serovar identification if isolated, polymerase chain reaction (PCR targeting outer membrane protein LipL32) testing and serology (Microscopic Agglutination Test; MAT; Table S2) were performed at Australia’s Leptospirosis Reference Laboratory in Brisbane, 1390 km away from Cairns Hospital [29]. If individuals had no chest imaging performed during their hospitalisation, they were not included in this study.

The patients’ EMRs were examined to collect demographic data and to identify any comorbidities (Table S3). Individuals less than 16 years of age were defined as children. The patients’ symptoms and clinical signs that were documented at their presentation to Cairns Hospital were also recorded. Laboratory data were collected from the Queensland statewide electronic database AUSLAB; these data included the values at presentation (either at the regional referring hospital or at Cairns Hospital) and the highest or, where relevant, lowest values during the patient’s hospitalisation.

Specialist radiologist reporting of any imaging was also collated from the health system’s electronic database. Individuals were said to have lung involvement if they had any new lung parenchymal changes (which were felt to represent acute changes by the reporting specialist radiologist) on this chest imaging. Pulmonary haemorrhage was defined as present if there was documented frank haemoptysis or if frank blood was present on tracheal aspirate. ARDS was classified using the Berlin definition based on the partial pressure of arterial oxygen: fraction of inspired oxygen (PaO_2_/FiO_2_) ratio where this was available [30]. As many individuals did not have an arterial blood gas collected during their hospitalisation the pulse oximetry oxygen saturation: fraction of inspired oxygen ratio (SpO_2_/FiO_2_) was also calculated [31]. Oliguria was defined as a documented urine output of less than 0.5 mL/kg/h.

The duration of symptoms prior to antibiotic therapy was noted. If patients developed pulmonary haemorrhage, required ICU admission, needed vasopressor support, required intubation and mechanical ventilation, needed renal replacement therapy (RRT) or required transfer to a quaternary centre for more specialised care, this was recorded. The administration of corticosteroids was documented, as was the agent that was chosen and the duration of this therapy. The total hospital length of stay and the length of stay in the ICU, where relevant, were recorded. All-cause 90-day mortality was documented.

### 2.1. Statistical Analysis

Data were de-identified and entered into a centralised electronic database (Microsoft Excel, Redmond, WA, USA) and analysed using statistical software (Stata version 18, College Station, TX, US). As many of the continuous variables had a non-parametric distribution these data are presented as the median and interquartile range (IQR). Groups were analysed using the Wilcoxon rank-sum test, the chi-squared test, Fisher’s exact test or logistic regression, where appropriate. All *p*-values were 2 sided and statistical significance was set at *p* < 0.05. If individuals were missing data, they were not included in analyses which evaluated those variables.

### 2.2. Ethical Approval

The study was conducted in accordance with the Declaration of Helsinki and approved by the Far North Queensland Human Research Ethics Committee (HREC/EX/2024/QCH/108994) on the 2 August 2024. As the retrospective data were de-identified and presented in an aggregated manner, the Committee waived the requirement for informed consent.

## 3. Results

There was a total of 111 individuals with laboratory-confirmed leptospirosis admitted to Cairns Hospital during the study period. Elements of the presentation, diagnosis, care and clinical course of the cohort have been reported previously [25]. There were 2/111 (2%) who had no chest imaging performed during their hospitalisation, leaving 109 individuals who were included in this analysis. Their median age of these 109 individuals was 39 (IQR: 24–56) years, 93/109 (85%) were male, and 6/109 (6%) were children. Most individuals (65/109, 60%) presented during the wet season; 83/109 (76%) were transferred to Cairns Hospital from a smaller regional health facility for escalation of care.

Leptospirosis PCR was performed on blood in 98/109 (89%) patients and was positive (*lipL*32 target detected) in 88/98 (90%); whole-blood culture for leptospirosis was performed in 47/109 (44%) and was positive in 29/47 (62%). Overall, PCR of blood or whole-blood culture was positive in 92/101 (91%) who had either of these tests performed. Acute serology testing by MAT was performed in 102/109 (94%) and was reactive in 44/102 (43%). Convalescent serology was collected and tested by MAT in 50/109 (46%). Convalescent serology was frequently performed to infer the infecting serovar, but it established the diagnosis of leptospirosis in 11/50 (22%). It was possible to infer the serovar in 62 individuals; *Leptospira interrogans* serovar Zanoni (24/62, 39%) and *L. interrogans* serovar Australis were the most common (12/62, 19%) (Table S4).

### 3.1. Lung Involvement

Overall, 62/109 (57%) had lung involvement; there was no difference in the demographic characteristics or the comorbidities of patients who had lung involvement and those who did not (Table 1). Notably, lung involvement was neither more common in individuals with chronic lung disease nor in those who were current smokers. Individuals with lung involvement had a longer duration of symptoms prior to the commencement of antibiotic therapy than individuals without lung involvement.

Among the 62 individuals with lung involvement, 27 (44%) had respiratory symptoms at presentation: 23 (37%) had a cough, 12 (19%) had haemoptysis and 11 (18%) complained of dyspnoea; 34/62 (54%) had abnormal chest auscultation at presentation (Table 2). On initial laboratory testing, individuals with lung involvement had greater renal impairment, a higher C-reactive protein and were more likely to have an abnormal troponin than individuals without lung involvement (Tables S5 and S6). The blood PCR or blood cultures were positive for leptospirosis in 57/62 (91%) of individuals with lung involvement in the cohort and in all 41 individuals with lung involvement who required ICU admission. Individuals with lung involvement were more likely to have infection with *L. interrogans* serovar Zanoni than individuals without lung involvement (Table 1).

**Figure 1:**
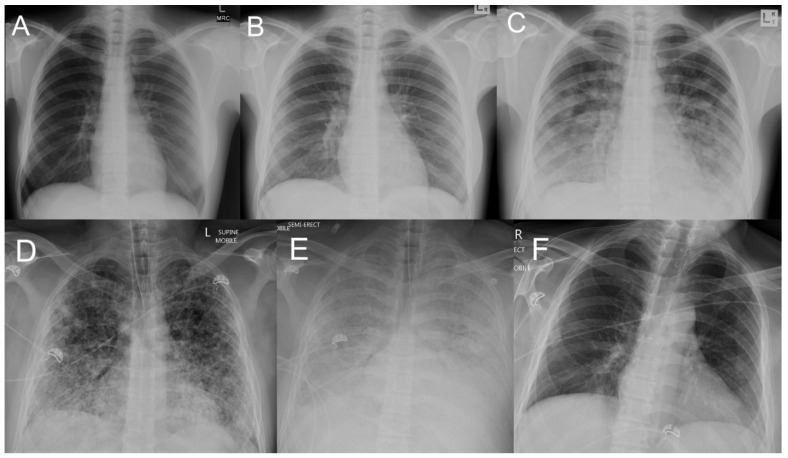
Illustrative chest X-ray (CXR) findings in individuals admitted to a referral hospital with leptospirosis in Far North Queensland, January 2015–June 2024, who had lung involvement. (**Panels A**–**C**): CXR imaging of an otherwise healthy 24-year-old female who presented with a 3-day history of fevers, but no respiratory symptoms and a normal chest X-ray (**Panel A**—day 1). She was commenced on ceftriaxone and azithromycin but remained febrile. A repeat CXR (**Panel B**—day 2) shows blunting of the left costophrenic angle without any focal parenchymal abnormality, but she developed dyspnoea and an oxygen requirement over the subsequent 48 h (**Panel C**–day 5) demonstrates the associated widespread patchy alveolar changes). With supportive care, she avoided an ICU admission, made a full recovery and was discharged after a 7-day hospitalisation. (**Panels D**–**F**): CXR imaging of an otherwise healthy 37-year-old male reporting a four-day history of cough, fevers and general malaise. At presentation, he had type I respiratory failure and required intubation in the Emergency Department before transfer to ICU. A CXR (**Panel D**—day 1) performed on arrival to Cairns Hospital revealed widespread bilateral patchy airspace consolidation. On day 2 of his admission, blood stained sputum was reported and a repeat CXR (**Panel E**) showed significant deterioration with diffuse airspace opacities consistent with pulmonary haemorrhage. Refractory hypoxia necessitated commencement of ECMO which he received for 20 days. (**Panel F**) 26 days after his presentation, shows nearly complete resolution of the lung changes. He was discharged after a total of 8 weeks in hospital.

**Figure 2:**
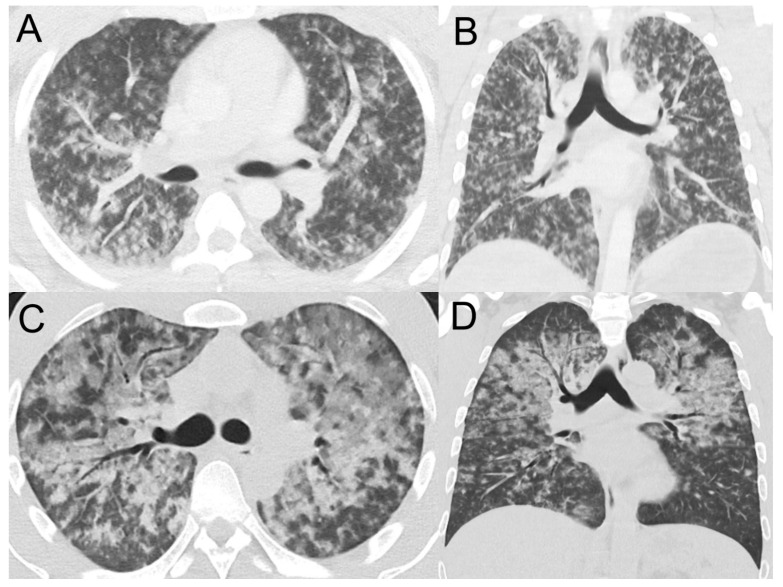
Illustrative computed tomography (CT) findings in individuals admitted to a referral hospital with leptospirosis in Far North Queensland, January 2015–June 2024, who had lung involvement. (**Panels A**,**B**): CT chest images ((**A**): axial and (**B**): coronal) of an otherwise healthy 30-year-old male who presented with 4 days of generalised malaise, fevers, and haematuria. On presentation he was haemodynamically stable and had no respiratory symptoms; however, within 8 h he deteriorated requiring supplemental oxygen and vasopressor support with noradrenaline. Images shortly after deterioration demonstrate diffuse alveolar opacities, ground glass opacities, and centrilobular nodules. The patient was admitted to ICU where he required intubation and mechanical ventilation. He made a full recovery and was discharged after a 20-day hospitalisation. (**Panels C**,**D**): CT chest images ((**C**): axial and (**D**): coronal) of an otherwise healthy 37-year-old male who presented with a 3-day history of cough, fevers, and dyspnoea. He was tachypnoeic at presentation but had a clear chest on auscultation, normal oxygen saturation on room air by pulse oximetry and a normal chest X-ray. Within 3 h of presentation the patient developed worsening respiratory distress and an oxygen requirement. At this time, the CT chest revealed extensive ground glass opacities with relative sparing of the lung bases, suggestive of diffuse pulmonary haemorrhage. He required an 8-day ICU admission for vasopressor support with noradrenaline and vasopressin but did not require intubation. He made a full recovery and was discharged from hospital after an 11-day admission.

The acute parenchymal changes on chest imaging that defined lung involvement were present on initial imaging in 36/62 (58%) and were present on subsequent imaging in another 26 (Table 3). These changes were often multilobar and opacities were frequently alveolar. The presence of multilobar or alveolar changes were associated with a more complicated disease course (Table 3). Nodular parenchymal changes were often present in the most critically ill patients (Figure 1 and Figure 2).

### 3.2. Pulmonary Haemorrhage

Pulmonary haemorrhage was diagnosed in 26/109 (24%). Individuals with pulmonary haemorrhage were more likely to be current smokers than individuals without pulmonary haemorrhage (14/26 (54%) versus 26/83 (31%), *p* = 0.04). There were no other demographic or clinical associations with the development of pulmonary haemorrhage. In particular, there was no association with the development of pulmonary haemorrhage and the presence of underlying lung disease or the infecting serovar. The median (IQR) duration of symptoms prior to the initiation of antibiotic therapy in the individuals who developed pulmonary haemorrhage was 5 (4–6) days versus 4 (3–5) days in the individuals who did not develop pulmonary haemorrhage, but this difference did not reach statistical significance (*p* = 0.10) (Table S7).

Among the 26 individuals with pulmonary haemorrhage, 16 (62%) had respiratory symptoms at presentation: 14 (54%) had a cough, 12 (46%) had haemoptysis and 8 (31%) complained of dyspnoea; 18 (69%) had abnormal chest auscultation at presentation. Individuals with pulmonary haemorrhage had lower SpO_2_/FiO_2_ ratios at presentation and were more likely to have oliguria and require vasopressor support than patients who did not have pulmonary haemorrhage. The other clinical and laboratory findings in the individuals who developed pulmonary haemorrhage are presented in Tables S8–S10. At presentation, individuals who developed pulmonary haemorrhage had greater renal impairment, a higher C-reactive protein and were more likely to have an abnormal troponin than individuals without lung involvement; 24/25 (96%) individuals with pulmonary haemorrhage who had PCR testing of blood were PCR positive and 7/15 (50%) who had blood cultures collected had a positive culture.

### 3.3. Clinical Course and Therapy

It was not possible to confirm the diagnosis of leptospirosis for several days and prior to this, patients tended to receive standard ward-based management guided by the patients’ physiological parameters. This care included antibiotic therapy, cautious crystalloid fluid therapy, and prompt initiation of additional supportive care—including vasopressors, RRT, mechanical ventilation and ECMO—as necessary, using standard indications [32]. Vasopressors were administered in accordance with clinical judgment titrated to achieve a mean arterial pressure of 65 mmHg. All patients received noradrenaline as first line vasopressor support; this was supplemented by vasopressin and adrenaline as necessary; inotropic support with milrinone or dobutamine was provided in the setting of significant left ventricular impairment demonstrated on echocardiogram. The median (IQR) duration of vasopressor support was 2 (1–3) days.

**Figure 3:**
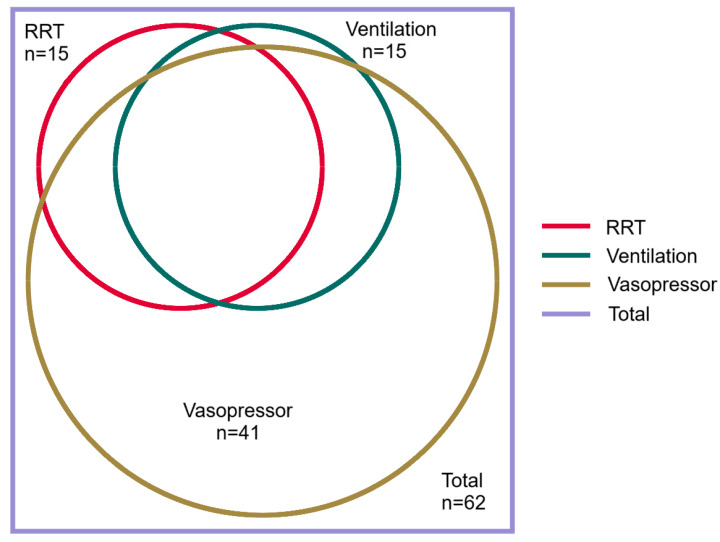
Venn diagram demonstrating the supportive care provided to the patients with laboratory confirmed leptospirosis and lung involvement in a referral hospital in Far North Queensland, January 2015 to June 2024. RRT: renal replacement therapy. There were 10/62 (16%) who received vasopressor support, RRT and mechanical ventilation; 5/62 (8%) who received vasopressor support and mechanical ventilation; 3/62 (5%) who received vasopressor support and RRT; 23/62 (37%) who received only vasopressor support and 2/62 (3%) who received only RRT. There were 19/62 (31%) individuals with lung involvement who did not receive vasopressor support, RRT or mechanical ventilation.

Individuals with lung involvement frequently had multi-organ dysfunction and were more likely to require ICU admission than individuals without lung involvement (41/62 (66%) versus 15/47 (32%), odds ratio (95% confidence interval): 4.17 (1.86–9.34), *p* < 0.001) (Figure 3 and Table 4). Arterial blood gas analysis was performed in 41/62 (50%) individuals with lung involvement; the median (IQR) nadir PaO_2_/FiO_2_ ratio was 236 (151–303); 30/62 (48%) satisfied the Berlin criteria for ARDS: 12/30 (40%) had mild, 15/30 (50%) had moderate, and 3/30 (10%) had severe ARDS. Intubation and mechanical ventilation were necessary in 15/62 (24%); all 15 of these individuals had lung involvement. The demographic, clinical and laboratory indices in individuals requiring mechanical ventilation are presented in Tables S11–S13.

Of the 109 patients, 107 (98%) received antibiotic therapy at some point in their hospitalisation. One patient (a patient with lung involvement) had a Jarisch–Herxheimer reaction, but this was managed successfully with simple supportive measures. A range of antibiotics were prescribed for a variety of durations, but doxycycline (taken by 81/109 (74%)) and ceftriaxone (received by 74/109 (68%)) were the agents prescribed most commonly. The two individuals who did not receive antibiotics were children (aged 7 and 11, respectively) who had the diagnosis of leptospirosis confirmed after the resolution of symptoms; neither had lung involvement. The details of the presentation, management and clinical course of the six children in the cohort are presented in Table S14.

Corticosteroids were prescribed in 19/109 (17%) and were prescribed more often in individuals with lung involvement (16/62 (26%) versus 3/47 (6%), *p* = 0.01). However, this corticosteroid therapy was usually intravenous hydrocortisone (typically at a dose of 50 mg four times a day) in the setting of refractory hypotension in individuals requiring vasopressor support; all 19 patients who received steroids received vasopressor support. Two individuals received high dose pulse methylprednisolone for severe pneumonitis, while one patient received dexamethasone in the setting of concurrent severe acute respiratory syndrome coronavirus 2 (SARS-CoV-2) infection. The median (IQR) duration of steroid therapy was 2 (1–4) days.

Two patients with refractory respiratory failure required evacuation to a quaternary centre to receive ECMO; one of these individuals also received intravenous tranexamic acid and nebulised adrenaline for pulmonary haemorrhage. Inhaled tranexamic acid was prescribed to 3 of the other 25 individuals with pulmonary haemorrhage. No patients received plasma exchange or plasmapheresis. All patients in the cohort were alive 90 days after presentation.

## 4. Discussion

The lungs are commonly affected in patients hospitalised with leptospirosis in tropical Australia, with radiological evidence of lung involvement identified in more than half of the patients in this cohort. This lung involvement is life-threatening: almost half of the patients with lung involvement satisfied criteria for ARDS and over 40% had pulmonary haemorrhage. Almost two-thirds of the patients with lung involvement required ICU admission, although importantly, lung involvement was commonly just one element of multi-organ dysfunction in these patients.

However, although leptospirosis was considered in the initial differential diagnosis of approximately 60% of the patients with lung involvement, the diagnosis could only be confirmed several days into the admission. Prior to this, patients tended to receive standard sepsis management which included antibiotic therapy, cautious fluid resuscitation with crystalloid, vasopressors (with low-dose corticosteroids as necessary) to maintain blood pressure and prompt initiation of additional supportive care—including mechanical ventilation, RRT and ECMO—guided by the patients’ physiological parameters and clinical trajectory [32]. Fortunately, with access to care in Australia’s well-resourced health system, every patient in the cohort—including the 57% with confirmed lung involvement—was alive 90 days after presentation.

Over 95% of the patients with lung involvement had no history of chronic lung disease. Indeed, over 40% of the patients with lung involvement were aged younger than 40 and had no comorbidity, highlighting the pathogen’s ability to cause life-threatening disease in otherwise well young people. The minority of patients with lung involvement during their illness had respiratory symptoms at presentation, only 55% had an abnormal respiratory examination, and only 58% had abnormal chest imaging, emphasising the importance of frequent clinical reassessment and a low threshold for repeat chest imaging in patients in whom leptospirosis is suspected, as clinical deterioration can be rapid [33].

The imaging findings in our cohort were similar to those identified in other series; multilobar and alveolar changes, in particular, were common and were associated with a more complicated disease course [34,35,36]. These radiological appearances would correlate with the histological findings of alveolar oedema and haemorrhage that are seen commonly in patients with fatal leptospirosis [37,38,39,40]. The precise pathophysiology of lung involvement in patients with leptospirosis remains incompletely understood, but human autopsy studies have demonstrated leptospiral antigen within endothelial cells of the interalveolar septa and attached to capillary endothelial cells, supporting the hypothesis that the organism exerts a locally destructive action on lung capillaries, leading to loss of vascular integrity and the alveolar oedema and haemorrhage which is seen in severe disease [33,38,39,41].

These clinicopathological correlation studies provide support for the management strategies employed in the patients with lung involvement in our cohort. Antibiotic therapy was prescribed to all but two individuals, both children without lung involvement. Some authors have expressed doubts about the incremental value of antibiotic therapy in patients with leptospirosis presenting later in their disease course and a Cochrane review—which emphasised that few quality studies have examined the issue—found little evidence to suggest benefit of antibiotic therapy in patients with the infection [5,12]. However, although patients with lung involvement in this cohort presented after a median of 5 days of symptoms, leptospiraemia was documented in 95% of those who had blood PCR performed or blood cultures collected (and in every patient with lung involvement who required ICU care). Indeed, the development of lung involvement was associated with delays in the administration of antibiotic therapy, echoing findings from other series and providing data to support the utility of antibiotic therapy in these patients [42,43].

In addition to the human autopsy studies demonstrating alveolar oedema—hypothesised to result from direct vascular injury—animal models of leptospirosis have documented decreased epithelial sodium channel (H-ENaC) protein expression and upregulation of the Na-K-2Cl cotransporter NKCC1 in the lungs which further impairs pulmonary fluid handling [37,38,39,44]. Although hypotension and AKI were common in the cohort, concerns about precipitating or exacerbating pulmonary oedema meant that fluid resuscitation was generally conservative in these patients, even before changes were apparent on chest imaging. Large fluid boluses were avoided in patients with hypotension or AKI who were instead managed using standard sepsis algorithms that included prompt vasopressor support to maintain a mean arterial pressure and, where necessary, RRT [32].

Pulmonary haemorrhage is the most common cause of death in patients with leptospirosis and even in ICUs with access to ECMO mortality can exceed 30% [20,39,40,45,46,47]. Pulmonary haemorrhage was identified in 26 individuals in our cohort and although this necessitated ECMO in two cases—and three others received tranexamic acid—the patients’ management was generally supportive and expectant, and outcomes were excellent. Although plasmapheresis is available locally, no patient had plasmapheresis or plasma exchange. There has been uncertainty about the contribution of disseminated intravascular coagulation (DIC) to the development of pulmonary haemorrhage; however, while patients with pulmonary haemorrhage had a lower nadir platelet count than patients without pulmonary haemorrhage, their coagulation studies did not suggest the presence of DIC [48]. It was notable that pulmonary haemorrhage was more common in current cigarette smokers, echoing findings from a New Caledonian series [43]. The explanation for this possible association is uncertain although it is plausible that smoking could lead to damage to the alveolar basement membrane, increasing the risk of haemorrhage [49,50]. The fact that over a third of the cohort were current cigarette smokers—compared to a current rate of 8.3% in Australians aged 14 or older—may have contributed to the high incidence of pulmonary haemorrhage in our study [51].

Many authors have suggested that a dysregulated and intense immune response plays an important role in the pathogenesis of severe disease [5,52,53]. This is the basis for the high dose corticosteroid therapy that has been proposed by some authors, particularly for patients with lung involvement [54]. However, while infiltration of alveolar spaces by monocytes and neutrophils occurs, inflammatory infiltrates are generally not prominent [38,39,40,55]. Although almost 85% of the patients in our cohort with lung involvement received corticosteroids, this was usually modestly dosed hydrocortisone that was prescribed for less than 72 h in the setting of hypotension requiring vasopressor support, rather than the high dose bolus methylprednisolone for three days (followed by oral corticosteroids for five-seven days) that has been used in some series [17,54]. The encouraging outcomes seen in our cohort suggest that higher dose corticosteroid therapy should not be considered as a routine intervention in patients with lung involvement, particularly given the risk of nosocomial infection, until definitive data from adequately powered randomised controlled trials is available to support this approach [56,57].

It is important to highlight that even in Australia’s well-resourced health system where there is access to a World Health Organization Collaborating Centre for Leptospirosis, the diagnosis of leptospirosis in our cohort was frequently not confirmed for 5–7 days, and in some cases it was a retrospective diagnosis [29]. The clinical and laboratory findings in patients with leptospirosis, while frequently suggestive, are often non-specific and it can be difficult to distinguish patients with leptospirosis from patients with other life-threatening infections and, indeed, non-communicable diseases [32,48,58,59,60]. Therefore, it is important not to institute specific interventions for the management of leptospirosis that fails to consider these other diagnoses or contains elements that may—either by omission or commission—increase the risk of deterioration. In this context the pragmatic clinician can be reassured that many elements of the care received by the patients with laboratory-confirmed leptospirosis in this cohort would be equally appropriate in patients with the other infectious diseases that may mimic it. These include early antibiotic therapy [32,61,62], a conservative approach to fluid resuscitation [63,64,65,66], cautious use of corticosteroids [67,68], and the early delivery of context dependent critical care support [32,69,70].

Although this study was able to examine the demographic, clinical, laboratory and radiological findings of patients with lung involvement complicating laboratory-confirmed leptospirosis in some detail, it has several limitations. The study’s retrospective nature meant that patients’ clinical assessment, investigations and management were not standardised. They occurred at different stages in the evolution of the disease and the reporting of comorbidities and clinical findings, relied on accurate documentation in the medical record. The study examined only patients managed in a referral hospital and therefore included patients with a more severe clinical phenotype, overestimating the proportion of cases that develop severe disease. The definition of lung involvement, while pragmatic, is imperfect. Although we used the imaging reports of a specialist radiologist, a plain chest X-ray may not identify early parenchymal changes and lung imaging was performed only once in a third of the cohort; this will tend to underestimate the true frequency of lung involvement [36]. The categorical definition of lung involvement lacked nuance; there is clearly a significant clinical difference between subtle, asymptomatic chest X-ray changes and multilobar alveolar changes causing respiratory failure. The definition of pulmonary haemorrhage was equally crude and required adequate documentation in the medical record. There is some geographical variation in the clinical presentation of leptospirosis, which some clinicians might suggest limits the applicability of our observations to other settings [16,71,72,73]. Infection with serovars from the Icterohemorrhagiae serogroup has been reported to be associated with lung involvement and pulmonary haemorrhage [33,73,74,75]. However, it was notable that serovars from the Pyrogenes serogroup (Zanoni and Robinsoni) and Australis serogroup (Australis), the most common serovars in the region, were also the most commonly identified serovars in patients with lung involvement and pulmonary haemorrhage in this cohort. These data support the contention that there may be limited correlation between particular serovars and the severity and clinical manifestations of leptospirosis [52,76,77].

It is important to highlight that the patients in our cohort were managed in Australia’s well-resourced, universal health system which limits the generalisability of some of our recommendations, particularly to the resource-limited settings where globally, most cases of leptospirosis are seen [1]. That said, we would argue that the clinical approach and management strategies which include prompt antibiotic therapy, conservative fluid resuscitation, efforts to exclude alternative diagnoses and the provision of additional supportive care—where this is available—are likely to be equally relevant in these locations [32,70]. Although we have presented the general approach to the management of patients with leptospirosis in the FNQ region, detailed quantitative data describing the different interventions (including precise volumes of fluid, the dosing of vasopressors, ventilation strategies, blood product support and the indications for mechanical ventilation, RRT and ECMO) have not been presented. They will be the focus of a manuscript that is in preparation. Separate reports will also examine the manifestations of leptospirosis in other organ systems in patients in the FNQ region.

Future prospective studies should examine how to expedite the diagnosis of leptospirosis in the resource-limited settings where leptospirosis is most frequently encountered [78]. These studies should also confirm that antibiotic therapy has a salutary effect on the evolution of severe leptospirosis and define simple, safe and effective fluid resuscitation protocols that can be supervised by less experienced health workers. The risks and benefits of different doses of corticosteroid therapy need to be defined in randomised, controlled trials in different clinical settings to determine if the higher doses used in observational studies have any advantage over the lower doses used in the contemporary management of the patient with sepsis [57,79,80]. Further examination of the apparent association between pulmonary haemorrhage and cigarette smoking may provide fresh insights into the pathophysiology of this life-threatening condition, while the utility of adjuvant therapies (including tranexamic acid and nebulised adrenaline) in its management should also be clarified [22,81]. Optimal thresholds for platelet and blood transfusion should also be defined for patients with pulmonary haemorrhage, recognising that they are frequently managed in settings where there is limited access to blood product support [1,3,82]

## 5. Conclusions

This study emphasises that life-threatening lung involvement can complicate leptospirosis in young and otherwise well individuals. The cohort had a median age of only 39 and almost 90% of the patients had no comorbidity. However, there was radiological evidence of lung involvement in 57% of the patients, which was frequently just one component of multi-organ dysfunction requiring ICU care. Pulmonary haemorrhage—which has a case-fatality rate that can exceed 50%—was present in almost a quarter of the cohort [83]. However, patients who develop these complications who are cared for in Australia’s well-resourced health system can have excellent outcomes using a contemporary standard approach to the management of undifferentiated infection and sepsis while a confirmed diagnosis of leptospirosis is awaited.

## Figures and Tables

**Table 1:** The demographics and comorbidities of individuals admitted to a referral hospital with leptospirosis in Far North Queensland, January 2015–June 2024, and the association of these factors with the development of lung involvement.

Variable	All n = 109	No Lung Involvement ^a^ n = 47	Lung Involvement ^a^ n = 62	*p*
Age (years) ^b^	38 (24–56)	33 (21–53)	42 (26–62)	0.07
Child (age < 16 years)	6 (6)	3 (6)	3 (5)	1.0
Male sex	93 (85)	39 (83)	54 (87)	0.55
Rural or remote residence ^c^	87 (80)	36 (77)	51 (81)	0.47
Wet season presentation	65 (60)	28 (60)	37 (60)	0.99
Leptospirosis in initial differential diagnosis	70 (64)	32 (68)	38 (61)	0.46
Duration of symptoms prior to antibiotic therapy (days) ^b^	4 (3–5)	3 (2–5)	5 (4–6)	0.001
Any comorbidity ^d^	13 (12)	6 (13)	7 (11)	1.0
Diabetes mellitus ^d^	2 (2)	1 (2)	1 (2)	1.0
Cardiac failure ^d^	3 (3)	2 (4)	1 (2)	0.58
Ischaemic heart disease ^d^	2 (2)	1 (2)	1 (2)	1.0
Chronic kidney disease ^d^	0	0	0	-
Chronic lung disease ^d^	5 (5)	2 (4)	3 (5)	1.0
Liver disease ^d^	5 (5)	3 (6)	2 (3)	0.65
Malignancy ^d^	2 (2)	0	2 (3)	0.51
Autoimmune disease ^d^	0	-	-	-
Immunosuppressed ^d^	0	-	-	-
Hazardous Alcohol use ^d^	29 (27)	13 (28)	16 (26)	0.83
Current smoker ^d^	40 (37)	14 (30)	26 (42)	0.19
PCR positive ^e^	88/98 (90)	33/40 (83)	55/58 (95)	0.09
Culture positive ^e^	30/47 (63)	15/21 (71)	14/26 (54)	0.25
Either PCR or culture positive ^e^	92/101 (91)	35/41 (85)	57/60 (95)	0.15
Serovar Zanoni ^e^	24/62 (39)	7/29 (24)	17/33 (52)	0.04
Serovar Australis ^e^	12/62 (19)	5/29 (17)	7/33 (21)	1.0

Number (percentage) or median (interquartile range) presented. PCR: polymerase chain reaction. ^a^ Lung involvement defined as any new acute changes in lung parenchyma on chest imaging at any stage during the patient’s hospitalisation. ^b^ Median (interquartile range presented). ^c^ Residing outside the city of Cairns. ^d^ As defined in Table S3. ^e^ PCR was performed in 98, culture was performed in 47 and 62 had a serovar identified.

**Table 2:** Symptoms and signs of individuals admitted to a referral hospital with leptospirosis in Far North Queensland, January 2015–June 2024, and the association of these factors with the development of lung involvement.

Variable	All n = 109	No Lung Involvement ^a^ n = 47	Lung Involvement ^a^ n = 62	*p*
***Subjective symptoms***				
Headache	79 (72)	36 (77)	43 (69)	0.40
Fevers	104 (95)	46 (98)	58 (94)	0.39
Rigors	40 (37)	16 (34)	24 (39)	0.62
Confusion	8 (7)	2 (4)	6 (10)	0.46
Fatigue	43 (39)	16 (34)	27 (44)	0.32
Abdominal pain	42 (39)	19 (40)	23 (37)	0.72
Myalgia	82 (75)	35 (74)	47 (76)	0.87
Arthralgia	46 (42)	21 (45)	25 (40)	0.65
Diarrhoea	39 (36)	13 (28)	26 (42)	0.12
Nausea/vomiting	73 (67)	33 (70)	40 (65)	0.53
Chest pain	9 (8)	3 (6)	6 (10)	0.73
Dyspnoea	16 (15)	5 (11)	11 (18)	0.30
Cough	32 (29)	9 (19)	23 (37)	0.04
URTI symptoms	15 (14)	7 (15)	8 (13)	0.79
Haemoptysis	12 (11)	0	12 (19)	0.001
Abnormal bleeding or bruising	11 (10)	4 (9)	7 (11)	0.75
***Objective examination findings***				
Hepatomegaly	11 (10)	3 (6)	8 (13)	0.35
Splenomegaly	0	0	0	-
Lymphadenopathy	6 (6)	1 (2)	5 (8)	0.23
Conjunctival suffusion	22 (20)	9 (19)	13 (21)	1.0
Skin rash	19 (17)	8 (17)	11 (18)	1.0
Abnormal chest auscultation	44 (40)	10 (21)	34 (55)	<0.001
***Vital signs at presentation***				
Oliguria ^b^	42 (39)	12 (26)	30 (48)	0.02
Temperature ° Celsius ^c^	37.1 (36.8–37.6)	37.2 (36.8–37.8)	37.1 (36.8–37.6)	0.62
Supplemental oxygen given	23 (21)	3 (6)	20 (32)	0.001
SpO_2_/FiO_2_	462 (450–471)	467 (457–471)	462 (296–467)	0.008
Respiratory rate	20 (18–24)	20 (18–24)	20 (18–25)	0.54
Heart rate	99 (80–115)	97 (78–107)	104 (83–116)	0.24
Systolic blood pressure ^c,d^	111 (100–124)	111 (99–124)	111 (104–124)	0.40
Vasopressors administered ^e^	42 (39)	11 (23)	31 (50)	0.005
Impaired consciousness	6 (6)	0	6 (10)	0.04
***Disease severity score***				
SPiRO score ^c,f^	1 (0–2)	1 (0–1)	2 (1–2)	0.001

Number (percentage) or median (interquartile range) presented. URTI: upper respiratory tract infection. SpO_2_/FiO_2_: oxygen saturation: fraction of inspired oxygen ratio. ^a^ Lung involvement defined as any new acute changes in lung parenchyma on chest imaging at any stage during the patient’s hospitalisation. ^b^ Documented urine output of less than 0.5 mL/kg/h. ^c^ Median (interquartile range) presented. ^d^ In the patients in whom vasopressor therapy was not initiated. ^e^ Vasopressors were administered in accordance with clinical judgement using standard algorithms [32]. All patients received noradrenaline as first line vasopressor support, supplemented by vasopressin and adrenaline as necessary; inotropic support with milrinone or dobutamine was provided in the setting of significant left ventricular impairment. ^f^ Three-point SPiRO score (Systolic blood Pressure < 100 mmHg, Respiratory auscultation abnormalities, Oliguria), each is awarded one point [24].

**Table 3:** Association between the findings on chest imaging and the subsequent clinical course of individuals admitted to a referral hospital with leptospirosis in Far North Queensland, January 2015–June 2024.

	All n = 109 n (%)	ICU Admission n = 56 Odds Ratio (95% CI)	*p*	Pulmonary Haemorrhage n = 26 Odds Ratio (95% CI)	*p*	Mechanical Ventilation n = 15 Odds Ratio (95% CI)	*p*
Multilobar changes	50 (46)	2.59 (1.19–5.54)	0.02	26.31 (5.78–119.71)	<0.001	22.56 (2.84–178.92)	<0.001
Alveolar changes	54 (50)	5.81 (2.55–13.27)	<0.001	21.20 (4.68–96.01)	<0.001	18.9 (2.39–149.71)	0.005
Only interstitial changes	8 (7)	0.29 (0.06–1.51)	0.14	0.43 (0.05–3.79)	0.45	0.89 (0.10–7.78)	0.91
No imaging changes	47 (43)	0.24 (0.11–0.54)	0.001	0.03 (0.00–0.25)	0.001	- ^a^	- ^a^

ICU: Intensive care unit. CI: confidence interval. ^a^ All 15 individuals requiring intubation and mechanical ventilation had lung involvement.

**Table 4:** Comparison of the clinical course of individuals admitted to a referral hospital with leptospirosis in Far North Queensland, January 2015–June 2024, stratified by the presence of lung involvement, pulmonary haemorrhage or a requirement for mechanical ventilation.

	All n = 109	No Lung Involvement n = 47	Lung Involvement n = 62	*p*	No Pulmonary Haemorrhage n = 83	Pulmonary Haemorrhage n = 26	*p*	No Mechanical Ventilation n = 94	Mechanical Ventilation n = 15	*p*
Time to antibiotics (days) ^a^	4 (3–5)	3 (2–5)	5 (4–6)	0.001	4 (3–5)	5 (4–6)	0.10	4 (3–5)	5 (4–6)	0.12
ICU admission	56 (51)	15 (32)	41 (66)	<0.001	37 (45)	19 (73)	0.01	41 (44)	15 (100)	<0.0001
ICU length of stay (days) ^a^	3 (2–5)	1 (1–3)	3 (2–6)	0.002	2 (1–3)	6 (3–11)	<0.0001	2 (1–3)	9 (6–15)	<0.0001
Received RRT	18 (17)	3 (6)	15 (24)	0.01	9 (11)	9 (35)	0.01	8 (9)	10 (67)	<0.0001
Received vasopressors ^b^	56 (51)	15 (32)	41 (66)	<0.001	37 (45)	19 (73)	0.01	41 (44)	15 (100)	<0.0001
Steroids prescribed	19 (17)	3 (6)	16 (26)	0.01	8 (10)	11 (42)	<0.0001	6 (6)	13 (87)	<0.0001
Received ECMO	2 (2)	0	2 (3)	0.51	0	2 (8)	0.06	0	2 (13)	0.02
Hospital length of stay (days) ^a^	6 (3–8)	4 (3–6)	7 (5–11)	<0.001	5 (3–7)	7 (6–14)	<0.0001	5 (3–7)	18 (10–21)	<0.0001
Died	0	0	0	-	0	0	-	0	0	-

Number (percentage) or median (interquartile range) presented. ICU: Intensive care unit; RRT: renal replacement therapy; ECMO: extracorporeal membranous oxygenation. ^a^ Median (interquartile range). ^b^ Vasopressor support was provided using standard algorithms and clinical judgement [32]. All patients received noradrenaline as their initial vasopressor, this was supplemented by vasopressin and adrenaline as necessary; inotropic support with milrinone or dobutamine was provided in the setting of significant left ventricular impairment.

## Data Availability

Data cannot be shared publicly because of the Queensland Public Health Act 2005. Data are available from the Far North Queensland Human Research Ethics Committee (contact via email: FNQ_HREC@health.qld.gov.au) for researchers who meet the criteria for access to confidential data.

## Supplementary Materials

The following supporting information can be downloaded at: https://www.mdpi.com/article/10.3390/tropicalmed10120333/s1, Figure S1. Map of Far North Queensland, Australia, showing catchment area for the current study. Table S1. Australian definition of laboratory confirmed leptospirosis. Table S2. Serovars in the *Leptospira* microscopic agglutination titre panel used at the Leptospirosis Reference Laboratory in Brisbane, Queensland. Table S3. Definitions used for comorbidities in the cohort. Table S4. The patients in whom a serovar was identified and the association with lung involvement, pulmonary haemorrhage or a requirement of intubation and mechanical ventilation (Serovars listed in bold were culture positive). Table S5. Haematological indices of individuals admitted to a referral hospital with leptospirosis in Far North Queensland, January 2015–June 2024, and the association of these factors with the development of lung involvement. Table S6. Biochemical indices of individuals admitted to a referral hospital with leptospirosis in Far North Queensland, January 2015–June 2024, and the association of these factors with the development of lung involvement. Table S7. The demographics and comorbidities of individuals admitted to a referral hospital with leptospirosis in Far North Queensland, January 2015–June 2024, and the association of these factors with the development of pulmonary haemorrhage. Table S8. The symptoms and signs at presentation of individuals admitted to a referral hospital with leptospirosis in Far North Queensland, January 2015–June 2024, and the association of these factors with the development of pulmonary haemorrhage. Table S9. Haematological indices of individuals admitted to a referral hospital with leptospirosis in Far North Queensland, January 2015–June 2024, and the association of these factors with the development of pulmonary haemorrhage. Table S10. Biochemical indices of individuals admitted to a referral hospital with leptospirosis in Far North Queensland, January 2015–June 2024, and the association of these factors with the development of pulmonary haemorrhage. Table S11. The symptoms and signs at presentation of individuals admitted to a referral hospital with leptospirosis in Far North Queensland, January 2015–June 2024, and the association of these factors with the requirement for intubation and mechanical ventilation. Table S12. Haematological indices of individuals admitted to a referral hospital with leptospirosis in Far North Queensland, January 2015–June 2024, and the association of these factors with the requirement for intubation and mechanical ventilation. Table S13. Biochemical indices of individuals admitted to a referral hospital with leptospirosis in Far North Queensland, January 2015–June 2024, and the association of these factors with the requirement for intubation and mechanical ventilation. Table S14. Presentation, management and clinical course of the six children admitted to a referral hospital with leptospirosis in Far North Queensland, January 2015–June 2024.

## Abbreviations

The following abbreviations are used in this manuscript:

AKI	Acute kidney injury
ARDS	Acute respiratory distress syndrome
DIC	Disseminated intravascular coagulation
ECMO	Extracorporeal membranous oxygenation
ICU	Intensive care unit
IQR	Interquartile range
RRT	Renal replacement therapy
