# Supplementary material for: Lung Involvement in Patients with Leptospirosis in Tropical Australia; Associations, Clinical Course and Implications for Management
Source: Trop Med Infect Dis. 2025 Nov 26;10(12):333. doi: 10.3390/tropicalmed10120333 (PMC12737544; doi:10.3390/tropicalmed10120333)
Supplement: Supplementary file 1 [file tropicalmed-10-00333-s001.zip › tropicalmed-3982971-supplementary.pdf]

Supplementary materials

Figure S1. Map of Far North Queensland, Australia, showing catchment area for the current study.

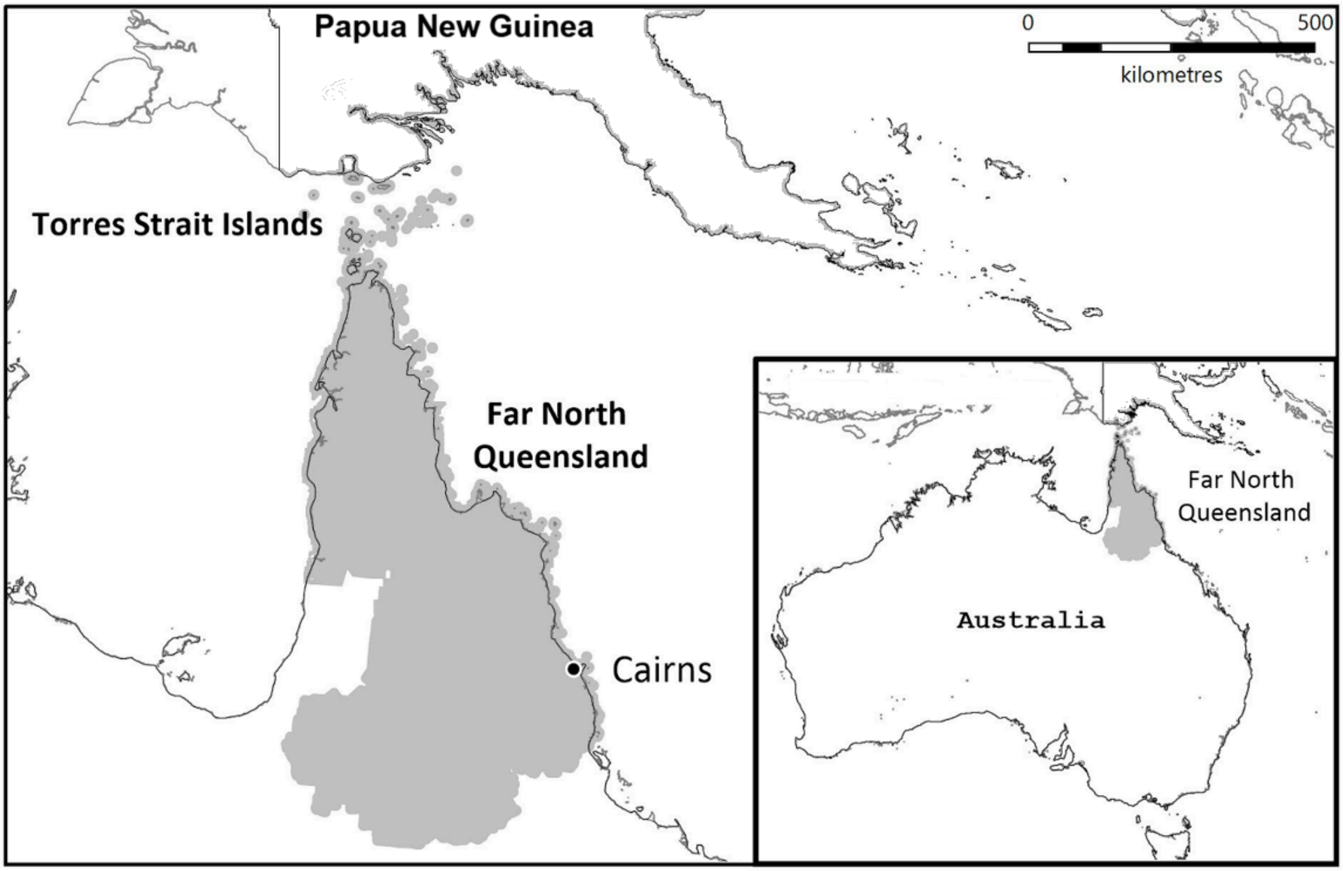

Table S1. Australian definition of laboratory confirmed leptospirosis [28].

To satisfy the definition of a laboratory confirmed diagnosis of infection with *Leptospira* spp. The individual must meet one or more of the following diagnostic criteria:
1. Isolation of pathogenic Leptospira spp. from a clinical specimen; or
2. Fourfold or greater increase of Leptospira agglutination titre by Microscopic Agglutination Test (MAT) between acute and convalescent phase serum obtained at least two weeks apart or
3. A single Leptospira agglutination titre by MAT greater than or equal to 400 in one or more serum specimens; or
4. Detection of Leptospira DNA by nucleic acid test (NAT) from a clinical specimen.

Table S2. Serovars in the *Leptospira* microscopic agglutination titre panel used at the Leptospirosis Reference Laboratory in Brisbane, Queensland [29].

**Species**	**Serogroup**	**Serovar**
Leptospira borgpetersenii	Ballum	Arborea
Leptospira interrogans	Australis	Australis
Leptospira interrogans	Bataviae	Bataviae
Leptospira kirschneri	Autumnalis	Bulgarica
Leptospira interrogans	Canicola	Canicola
Leptospira weilii	Celledoni	Celledoni
Leptospira interrogans	Icterohaemorrhagiae	Copenhageni
Leptospira kirschneri	Cynopteri	Cynopteri
Leptospira interrogans	Djasiman	Djasiman
Leptospira kirschneri	Grippotyphosa	Grippotyphosa
Leptospira borgpetersenii	Sejroe	Hardjo (subtype Hardjobovis)
Leptospira borgpetersenii	Javanica	Javanica
Leptospira interrogans	Hebdomadis	Kremastos
Leptospira interrogans	Sejroe	Medanensis
Leptospira noguchii	Panama	Panama
Leptospira interrogans	Pomona	Pomona
Leptospira interrogans	Pyrogenes	Robinsoni
Leptospira santarosai	Shermani	Shermani
Leptospira interrogans	Mini	Szwajizak
Leptospira borgpetersenii	Tarassovi	Tarassovi
Leptospira weilii	Tarassovi	Topaz
Leptospira interrogans	Pyrogenes	Zanoni

Table S3. Definitions used for comorbidities in the cohort [25].

Comorbidity		Definition used
If documented in patient's medical history recorded by the admitting doctor (categorical)	Diabetes mellitus	Documented in history, HbA1c > 6.5%, or on diabetic treatment
	Chronic Cardiac Failure	Documented in history, on heart failure therapy, or an echocardiogram demonstrating LVEF< 50%)
	Ischaemic heart disease	Documented in history as having a myocardial infarction or coronary artery bypass graft
	Chronic kidney disease	Documented in history or baseline eGFR <90 $mL/min/1.73m^2$
	Chronic lung disease	Documented in history; included interstitial lung disease, chronic obstructive pulmonary disease, bronchiectasis, cystic fibrosis, asthma or any other disease requiring therapy
	Chronic liver disease	Documented as having cirrhosis
	Active malignancy	Documented in history
	Autoimmune condition	Documented in history - any condition documented with –or without – immunomodulatory therapy
	Immunosuppression	If patient was taking any medications to suppress their immune system including regular corticosteroids, other immunosuppressants or immunomodulatory therapy
	Hazardous alcohol intake	>10 standard drinks per week or >4 standard drinks in one day at least once/month
	Smoker	Smoking tobacco on a regular basis

HbA1c: glycosylated haemoglobin; LVEF: left ventricular ejection fraction; eGFR: estimated glomerular filtration rate.

Table S4. The patients in whom a serovar was identified and the association with lung involvement, pulmonary haemorrhage or a requirement of intubation and mechanical ventilation (Serovars listed in bold were culture positive).

Study number	Age, gender	Serovar	Lung involvement	Pulmonary haemorrhage	Mechanical ventilation
16	67M	Zanoni	Yes	-	Yes
19	66M	**Zanoni**	Yes	Yes	-
22	63M	**Zanoni**	Yes	-	-
34	53M	Zanoni	Yes	-	Yes
42	45M	**Zanoni**	Yes	-	-
45	45M	**Zanoni**	-	-	-
47	45M	**Zanoni**	Yes	-	-
59	37M	Zanoni	Yes	Yes	Yes
60	38M	Zanoni	Yes	-	-
67	31M	**Zanoni**	Yes	Yes	-
68	25M	Zanoni	Yes	Yes	-
73	30 M	**Zanoni**	-	-	-
75	24F	**Zanoni**	Yes	-	-
81	27M	**Zanoni**	Yes	-	-
84	27M	**Zanoni**	-	-	-
87	24M	**Zanoni**	-	-	-
92	20M	**Zanoni**	Yes	Yes	-
93	20M	Zanoni	Yes	Yes	-
102	17M	Zanoni	Yes	-	-
103	11M	Zanoni	-	-	-
104	18M	Zanoni	Yes	-	-
106	16M	**Zanoni**	-	-	-
111	7F	Zanoni	-	-	-
38	45M	Zanoni, Robinsoni	Yes	Yes	Yes
11	64M	**Australis**	Yes	Yes	Yes
12	70F	**Australis**	Yes	-	-
23	61M	Australis	Yes	-	-
25	59M	Australis	Yes	-	-
29	57M	Australis	-	-	-
53	42F	Australis	Yes	-	-
55	40M	Australis	-	-	-
57	39F	**Australis**	-	-	-
91	26M	Australis	-	-	-
95	16M	Australis	-	-	-
72	30M	**Australis**	Yes	Yes	Yes
110	13M	Australis	Yes	Yes	Yes
21	59M	**Robinsoni**	Yes	-	Yes
32	54F	**Robinsoni**	-	-	-
33	53M	**Robinsoni**	-	-	-
36	42F	Robinsoni	Yes	Yes	Yes
61	31M	**Robinsoni**	Yes	Yes	-

64	27M	**Robinsoni**	-	-	-
13	68M	Arborea	-	-	-
39	45M	**Arborea**	Yes	-	-
63	33M	**Arborea**	-	-	-
79	23M	**Arborea**	-	-	-
96	16F	Arborea	-	-	-
105	16M	**Arborea**	-	-	-
18	66F	Copenhageni	-	-	-
54	35F	Copenhageni	-	-	-
44	45M	Copenhageni, Kremastos	-	-	-
40	45M	**Kremastos**	-	Yes	-
76	26M	**Kremastos**	-	-	-
50	43F	Kremastos, Djasiman	Yes	-	-
83	25M	Bulgarica	-	-	-
100	18M	Celledoni	-	-	-
107	16M	Cynopteri	Yes	-	-
4	71M	Hardjo	Yes	Yes	Yes
37	42M	Javanica	Yes	Yes	-
35	49M	Pomona	-	-	-
77	28M	**Topaz**	-	-	-
8	68M	Topaz, Batavaie, Djasiman	Yes	-	-

M: Male F: Female

Table S5. Haematological indices of individuals admitted to a referral hospital with leptospirosis in Far North Queensland, January 2015–June 2024, and the association of these factors with the development of lung involvement.

Variable	Reference range [a]	Number with data	All n=109	No lung involvement n=47	lung involvement n=62	P
Haemoglobin initial	115 - 160 g/dL	109	133 (122-147)	140 (129-151)	131 (120-142)	0.02
Haemoglobin lowest	115 - 160 g/dL	109	112 (98-123)	120 (107-127)	105 (89-118)	0.0001
White cell initial	4.0 - 11.0 x10^9/L	109	9.3 (6.9-12.1)	9.3 (7.1-11.6)	9.2 (6.6-12.4)	0.86
White cell highest	4.0 - 11.0 x10^9/L	109	13.1 (9.6-18.4)	11.1 (8.4-14.5)	15.2 (11.2-21.3)	0.001
Platelet initial	140 - 400 x10^9/L	109	114 (70-168)	147 (92-195)	87 (46-141)	0.0001
Platelet count lowest	140 - 400 x10^9/L	109	82 (31-121)	112 (76-137)	55 (24-98)	0.0001
Neutrophil initial	2.0 - 8.0 x10^9/L	109	8.2 (5.5-10.7)	8.4 (5.2-10.6)	8.2 (5.5-11.0)	0.91
Neutrophil highest	2.0 - 8.0 x10^9/L	109	11.1 (8.3-14.6)	10.0 (6.5-12.7)	11.9 (9.5-18.5)	0.006
Lymphocyte initial	1.0 - 4.0 x10^9/L	109	0.5 (0.3-0.7)	0.6 (0.3-0.8)	0.5 (0.3-0.6)	0.02
Lymphocyte lowest	1.0 - 4.0 x10^9/L	109	0.3 (0.2-0.5)	0.3 (0.2-0.6)	0.3 (0.2-0.4)	0.08
INR initial	0.9 - 1.2	83	1.1 (1.1-1.3)	1.1 (1.1-1.3)	1.1 (1.1-1.2)	0.60
INR highest	0.9 - 1.2	83	1.2 (1.1-1.3)	1.2 (1.1-1.3)	1.2 (1.1-1.3)	1.0
APTT initial	25 - 38 seconds	83	31 (28-34)	32 (29-34)	31 (28-34)	0.49
APTT highest	25 - 38 seconds	83	32 (30-36)	32 (29-35)	33 (30-38)	0.18

The median (interquartile range) is presented. INR: International normalised ratio. APTT: Activated partial thromboplastin time

[a] Queensland public hospital laboratory reference ranges

Table S6. Biochemical indices of individuals admitted to a referral hospital with leptospirosis in Far North Queensland, January 2015–June 2024, and the association of these factors with the development of lung involvement.

Variable	Reference range [a]	Number with data	All n=109	No lung involvement [a] n=47	Lung involvement [a] n=62	P
Initial serum sodium	135 - 145 mmol/L	109	133 (129-135)	134 (130-136)	132 (128-135)	0.11
Lowest serum sodium	135 - 145 mmol/L	109	132 (129-135)	133 (130-135)	131 (127-134)	0.001
Initial serum potassium	3.5 - 5.2 mmol/L	109	3.7 (3.4-4.0)	3.8 (3.4-4.0)	3.6 (3.4-3.8)	0.14
Highest serum potassium	3.5 - 5.2 mmol/L	109	4.4 (4.0-4.9)	4.3 (4.0-4.8)	4.6 (4.1-4.9)	0.13
eGFR initial	>90 mL/min/1.73 m^2	97	64 (25-90)	77 (38-90)	44 (23-77)	0.01
eGFR lowest	>90 mL/min/1.73 m^2	97	38 (14-78)	64 (21-90)	20 (12-55)	0.001
Initial serum creatinine	45 - 90 µmol/L	109	113 (88-205)	95 (82-150)	146 (96-258)	0.003
Highest serum creatinine	45 - 90 µmol/L	109	179 (102-382)	109 (92-229)	270 (144-484)	0.0001
Initial serum bicarbonate	22 - 32 mmol/L	109	23 (21-25)	23 (22-25)	23 (21-25)	0.48
Lowest serum bicarbonate	22 - 32 mmol/L	109	20 (17-22)	21 (19-22)	19 (17-22)	0.04
Initial serum total bilirubin	<20 µmol/L	109	18 (12-28)	15 (10-23)	20 (14-32)	0.03
Highest serum total bilirubin	<20 µmol/L	109	26 (19-48)	20 (13-45)	32 (23-51)	0.002
Initial serum ALT	<34 IU/ml	109	68 (27-115)	46 (25-96)	80 (27-121)	0.09
Highest serum ALT	<34 IU/ml	109	121 (68-208)	109 (57-174)	136 (92-228)	0.14
Initial serum AST	<31 IU/ml	109	63 (34-135)	56 (31-83)	95 (39-146)	0.10
Highest serum AST	<31 IU/ml	109	131 (74-210)	102 (66-195)	145 (80-257)	0.10
Initial serum GGT	<38 IU/ml	109	51 (22-120)	42 (19-93)	61 (25-164)	0.07
Highest serum GGT	<38 IU/ml	109	135 (70-235)	127 (51-217)	140 (77-310)	0.21
Initial serum SAP	30 - 110 IU/ml	109	98 (67-171)	87 (64-149)	99 (70-178)	0.15
Highest serum SAP	30 - 110 IU/ml	109	146 (109-208)	137 (97-184)	164 (110-221)	0.13
Initial serum CK	34-145 IU/ml	82	281 (104-1020)	170 (82-1020)	349 (118-1003)	0.33
Highest serum CK	34-145 IU/ml	82	350 (114-1020)	212 (82-1020)	401 (126-1080)	0.25
Initial serum CRP	<5 mg/L	105	187 (138-287)	156 (101-235)	234 (161-323)	0.004
Highest serum CRP	<5 mg/L	105	227 (159-323)	192 (138-289)	254 (182-355)	0.007
Initial serum lactate	0.5 - 2.2 mmol/L	104	1.5 (1.1-2.3)	1.5 (1.0-2.0)	1.7 (1.2-2.3)	0.18
Highest serum lactate	0.5 - 2.2 mmol/L	104	2.0 (1.4-2.7)	1.9 (1.3-2.4)	2.0 (1.4-3.4)	0.14
Elevated initial serum troponin [b]	-	109	25 (23%)	4 (9)	21 (34)	0.002
Elevated serum troponin during hospitalisation [b]	-	109	29 (26%)	5 (11)	24 (39)	0.001

The median (interquartile range) or the absolute number (percentage) is presented. eGFR: estimated glomerular filtration rate; ALT: alanine aminotransferase; AST: aspartate aminotransferase; GGT: gamma glutamyl transferase; SAP: serum alkaline phosphatase; CK: creatinine kinase; CRP: C-reactive protein.

[a] Queensland public hospital laboratory reference ranges.

[b] Troponin is expressed as a categorical variable as the Beckman Coulter assay for troponin I (normal reference range <0.040 µg/L) was replaced by the Siemens Atellica assay (reference range <20 ng/L normal for men; <10 ng/L normal for women) during the study perio

Table S7. The demographics and comorbidities of individuals admitted to a referral hospital with leptospirosis in Far North Queensland, January 2015–June 2024, and the association of these factors with the development of the development of pulmonary haemorrhage.

Variable	All n=109	No pulmonary haemorrhage [a] n=83	Pulmonary haemorrhage [a] n=26	P
Age (years)	38 (24-56)	40 (24-56)	37 (25-51)	0.87
Child (age <16 years)	6 (6)	4 (5)	2 (8)	0.63
Male sex	93 (85)	69 (83)	24 (92)	0.35
Remote residence	16 (15)	10 (12)	6 (23)	0.21
Rural or remote residence	87 (80)	64 (77)	23 (88)	0.27
Wet season presentation	65 (60)	55 (66)	10 (38)	0.01
Leptospirosis in initial differential diagnosis	70 (64)	53 (64)	17 (65)	0.89
Duration of symptoms prior to antibiotic therapy (days)	4 (3-5)	4 (3-5)	5 (4-6)	0.09
Any comorbidity [b]	13 (12)	12 (14)	1 (4)	0.18
Diabetes mellitus [b]	2 (2)	2 (2)	0	1.0
Cardiac failure [b]	3 (3)	3 (4)	0	1.0
Ischaemic heart disease [b]	2 (2)	2 (2)	0	1.0
Chronic kidney disease [b]	0	-	-	-
Lung disease [b]	5 (5)	5 (6)	0	0.34
Liver disease [b]	5 (5)	5 (6)	0	0.34
Malignancy [b]	2 (2)	1 (1)	1 (4)	0.42
Autoimmune disease [b]	0	-	-	-
Immunosuppressed [b]	0	-	-	-
Hazardous Alcohol use [b]	29 (27)	24 (29)	5 (19)	0.44
Smoker [b]	40 (37)	26 (31)	14 (54)	0.04
PCR positive [c]	88/98 (90)	64/73 (88)	24/25 (96)	0.44
Culture positive [c]	29/47 (62)	22/33 (67)	7/14 (50)	0.34
Serovar Zanoni	24/62 (39)	16/47 (34)	8/15 (53)	0.34
Serovar Australis	12/62 (19)	9/47 (19)	3/15 (20)	1.0

Number (percentage) presented

[a] Lung involvement is defined as any new acute changes in lung parenchyma on chest imaging

[b] As defined in Table S2.

[c] PCR was performed in 98, culture was performed in 47 and 62 had a serovar identified.

Table S8. The symptoms and signs at presentation of individuals admitted to a referral hospital with leptospirosis in Far North Queensland, January 2015–June 2024, and the association of these factors with the development of pulmonary haemorrhage.

Variable	Number with data	All n=109	No pulmonary haemorrhage [a] n=83	Pulmonary haemorrhage [a] n=26	P
Subjective symptoms					
Headache	109	79 (72)	63 (76)	16 (62)	0.15
Fevers	109	104 (95)	80 (96)	24 (92)	0.59
Rigors	109	40 (37)	32 (39)	8 (31)	0.64
Confusion	109	8 (7)	6 (7)	2 (8)	1.0
Fatigue	109	43 (39)	30 (36)	13 (50)	0.21
Abdominal pain	109	42 (39)	34 (41)	8 (31)	0.35
Myalgia	109	82 (75)	62 (75)	20 (77)	1.0
Arthralgia	109	46 (42)	32 (39)	14 (54)	0.17
Diarrhoea	109	39 (36)	27 (33)	12 (46)	0.21
Nausea/vomiting	109	73 (67)	56 (67)	17 (65)	1.0
Chest pain	109	9 (8)	7 (8)	2 (8)	1.0
Dyspnoea	109	16 (15)	8 (10)	8 (31)	0.02
Cough	109	32 (29)	18 (21)	14 (54)	0.002
URTI symptoms	109	15 (14)	11 (13)	4 (15)	0.75
Haemoptysis	109	12 (11)	0	12 (46)	<0.001
Abnormal bleeding or bruising	109	11 (10)	6 (7)	5 (19)	0.13
Objective examination findings					
Hepatomegaly	109	11 (10)	7 (8)	4 (15)	0.29
Splenomegaly	109	0	0	0	-
Lymphadenopathy	109	6 (6)	5 (6)	1 (4)	1.0
Conjunctival suffusion	109	22 (20)	17 (20)	5 (19)	1.0
Skin rash	109	19 (17)	15 (18)	4 (15)	1.0
Abnormal chest auscultation	109	44 (40)	26 (31)	18 (69)	0.001
Vital signs at presentation					
Oliguria [b]	109	42 (39)	25 (30)	17 (A65)	0.002
Temperature ° Celsius	109	37.1 (36.8-37.6)	37.1 (36.8-37.6)	37.2 (36.8-37.7)	0.92
Supplemental oxygen given	109	23 (21)	11 (13)	12 (46)	<0.001
SpO_2/FiO_2	109	462 (450-471)	467 (457-471)	455 (197-467)	0.001
Respiratory rate	109	20 (18-24)	20 (17-22)	20 (20-30)	0.02
Heart rate	109	99 (80-115)	96 (78-111)	110 (95-122)	0.01
Systolic blood pressure [c]	109	107 (96-120)	106 (96-120)	108 (94-121)	0.90
Vasopressors	109	42 (39)	27 (33)	15 (58)	0.02
Impaired consciousness	109	6 (6)	1 (1)	5 (19)	0.003
Disease severity score					
SPiRO score [d]	109	1 (0-2)	1 (0-2)	2 (1-3)	<0.001

Number (percentage) or median (interquartile range) presented

[a] Pulmonary haemorrhage defined as documented frank haemoptysis or frank blood present on tracheal aspirate

[b] Documented urine output of less than 0.5 mL/kg/hour

[c] In the patients in whom vasopressor therapy was not initiated

[d] Three-point SPiRO score (Systolic blood Pressure < 100 mmHg, Respiratory auscultation abnormalities, Oliguria), each is awarded one point [24]

Table S9. Haematological indices of individuals admitted to a referral hospital with leptospirosis in Far North Queensland, January 2015–June 2024, and the association of these factors with the development of pulmonary haemorrhage.

Variable	Reference range [a]	Number with data	All n=109	No pulmonary haemorrhage n=83	Pulmonary haemorrhage n=26	P
Haemoglobin initial	115 - 160 g/dL	109	133 (122-147)	135 (123-149)	126 (117-135)	0.008
Haemoglobin lowest	115 - 160 g/dL	109	112 (98-123)	117 (104-125)	91 (77-111)	<0.0001
White cell initial	4.0 - 11.0 $x10^9$/L	109	9.3 (6.9-12.1)	9.0 (6.7-11.5)	10.5 (8.1-13.3)	0.10
White cell highest	4.0 - 11.0 $x10^9$/L	109	13.1 (9.6-18.4)	11.3 (8.2-15.4)	18.9 (15.2-24.2)	<0.0001
Platelet initial	140 - 400 $x10^9$/L	109	114 (70-168)	131 (80-180)	59 (35-110)	0.0001
Platelet count lowest	140 - 400 $x10^9$/L	109	82 (31-121)	95 (56-126)	33 (21-62)	0.0004
Neutrophil initial	2.0 - 8.0 $x10^9$/L	109	8.2 (5.5-10.7)	8.1 (5.4-10.4)	9.4 (7.1-12.1)	0.08
Neutrophil highest	2.0 - 8.0 $x10^9$/L	109	11.1 (8.3-14.6)	10.2 (6.4-12.7)	15.6 (11.7-20.5)	<0.0001
Lymphocyte initial	1.0 - 4.0 $x10^9$/L	109	0.5 (0.3-0.7)	0.6 (0.3-0.7)	0.4 (0.3-0.6)	0.16
Lymphocyte lowest	1.0 - 4.0 $x10^9$/L	109	0.3 (0.2-0.5)	0.3 (0.2-0.5)	0.3 (0.2-0.4)	0.71
INR initial	0.9 - 1.2	83	1.1 (1.1-1.3)	1.1 (1.1-1.3)	1.1 (1.1-1.2)	0.57
INR highest	0.9 - 1.2	83	1.2 (1.1-1.3)	1.1 (1.1-1.3)	1.2 (1.1-1.3)	0.12
APTT initial	25 - 38 seconds	83	31 (28-34)	31 (29-34)	31 (28-36)	0.68
APTT highest	25 - 38 seconds	83	32 (30-36)	32 (30-35)	34 (31-44)	0.04

The median (interquartile range) is presented. INR: International normalised ratio. APTT: Activated partial thromboplastin time

[a] Queensland public hospital laboratory reference ranges

Table S10. Biochemical indices of individuals admitted to a referral hospital with leptospirosis in Far North Queensland, January 2015–June 2024, and the association of these factors with the development of pulmonary haemorrhage.

Variable	Reference range [a]	Number with data	All n=109	No pulmonary haemorrhage n=83	Pulmonary haemorrhage n=26	P
Initial serum sodium	135- 145 mmol/L	109	133 (129-135)	134 (130-136)	131 (127-134)	0.03
Lowest serum sodium	135- 145 mmol/L	109	132 (129-135)	132 (129-135)	130 (125-132)	0.007
Initial serum potassium	3.5 - 5.2 mmol/L	109	3.7 (3.4-4.0)	3.7 (3.4-4.0)	3.5 (3.3-3.9)	0.15
Highest serum potassium	3.5 - 5.2 mmol/L	109	4.4 (4.0-4.9)	4.3 (4.0-4.8)	4.8 (4.3-5.3)	0.004
eGFR initial	>90 mL/min/1.73 m^2	97	64 (25-90)	73 (29-90)	46 (22-68)	0.05
eGFR lowest	>90 mL/min/1.73 m^2	97	38 (14-78)	45 (16-90)	19 (11-43)	0.01
Initial serum creatinine	45 - 90 µmol/L	109	113 (88-205)	104 (84-188)	165 (106-277)	0.003
Highest serum creatinine	45 - 90 µmol/L	109	179 (102-382)	165 (95-342)	308 (181-484)	0.001
Initial serum bicarbonate	22 - 32 mmol/L	109	23 (21-25)	24 (22-25)	22 (20-25)	0.13
Lowest serum bicarbonate	22 - 32 mmol/L	109	20 (17-22)	20 (17-22)	19 (16-21)	0.16
Initial serum bilirubin	<20 µmol/L	109	18 (12-28)	17 (11-26)	22 (14-32)	0.09
Highest serum bilirubin	<20 µmol/L	109	26 (19-48)	24 (16-45)	40 (25-63)	0.005
Initial serum ALT	<34 IU/ml	109	68 (27-115)	55 (27-109)	77 (27-121)	0.41
Highest serum ALT	<34 IU/ml	109	121 (68-208)	120 (68-208)	129 (84-216)	0.58
Initial serum AST	<31 IU/ml	109	63 (34-135)	59 (34-115)	101 (34-173)	0.17
Highest serum AST	<31 IU/ml	109	131 (74-210)	115 (71-200)	171 (101-257)	0.07
Initial serum GGT	<38 IU/ml	109	51 (22-120)	46 (21-150)	61 (25-107)	0.44
Highest serum GGT	<38 IU/ml	109	135 (70-235)	137 (68-261)	135 (69-198)	0.77
Initial serum SAP	30 - 110 IU/ml	109	98 (67-171)	96 (64-169)	100 (66-198)	0.46
Highest serum SAP	30 - 110 IU/ml	109	146 (109-208)	145 (112-208)	166 (78-221)	0.93
Initial serum CK	34-145 IU/ml	82	281 (104-1020)	209 (102-737)	682 (198-1835)	0.01
Highest serum CK	34-145 IU/ml	82	350 (114-1020)	215 (105-771)	965 (27-2780)	0.004
Initial serum CRP	<5 mg/L	105	187 (138-287)	178 (136-283)	229 (166-316)	0.26
Highest serum CRP	<5 mg/L	105	227 (159-323)	216 (157-314)	254 (178-353)	0.24
Initial serum lactate	0.5 - 2.2 mmol/L	104	1.5 (1.1-2.3)	1.5 (1.1-1.9)	2.0 (1.2-3.2)	0.03
Highest serum lactate	0.5 - 2.2 mmol/L	104	2.0 (1.4-2.7)	1.9 (1.3-2.4)	2.5 (1.6-6.6)	0.02
Elevated initial serum troponin [c]	-	109	25 (23%)	16 (19)	9 (35)	0.12
Elevated serum troponin during hospitalisation [c]	-	109	29 (26%)	19 (23)	10 (38)	0.12

The median (interquartile range) or the absolute number (percentage) is presented. ALT: alanine aminotransferase; AST: aspartate aminotransferase; GGT: gamma glutamyl transferase; SAP: serum alkaline phosphatase; CK: creatinine kinase; CRP: C-reactive protein.

[a] Queensland public hospital laboratory reference ranges. [b] Troponin is expressed as a categorical variable as the Beckman Coulter assay for troponin I (normal reference range <0.040 µg/L) was replaced by the Siemens Atellica assay (reference range <20 ng/L normal for men; <10 ng/L normal for women) during the study period

Table S11. The symptoms and signs at presentation of individuals admitted to a referral hospital with leptospirosis in Far North Queensland, January 2015–June 2024, and the association of these factors with the requirement for intubation and mechanical ventilation.

Variable	Number with data	All n=109	Not intubated n=94	Intubated n=15	P
Subjective symptoms					
Headache	109	79 (72)	70 (74)	9 (60)	0.35
Fevers	109	104 (95)	90 (96)	14 (93)	0.53
Rigors	109	40 (37)	38 (40)	2 (13)	0.048
Confusion	109	8 (7)	6 (6)	2 (13)	0.30
Fatigue	109	43 (39)	35 (37)	8 (53)	0.27
Abdominal pain	109	42 (39)	37 (39)	5 (33)	0.78
Myalgia	109	82 (75)	72 (77)	10 (67)	0.52
Arthralgia	109	46 (42)	39 (41)	7 (47)	0.78
Diarrhoea	109	39 (36)	31 (33)	8 (53)	0.15
Nausea/vomiting	109	73 (67)	68 (72)	5 (33)	0.006
Chest pain	109	9 (8)	9 (10)	0	0.36
Dyspnoea	109	16 (15)	12 (13)	4 (27)	0.23
Cough	109	32 (29)	27 (29)	5 (33)	0.76
URTI symptoms	109	15 (14)	13 (14)	2 (13)	1.0
Haemoptysis	109	12 (11)	10 (11)	2 (13)	0.67
Abnormal bleeding or bruising	109	11 (10)	9 (10)	2 (13)	0.65
Objective examination findings					
Hepatomegaly	109	11 (10)	11 (12)	0	0.36
Splenomegaly	109	0	-	-	-
Lymphadenopathy	109	6 (6)	6 (6)	0	0.59
Conjunctival suffusion	109	22 (20)	21 (22)	1 (7)	0.30
Skin rash	109	19 (17)	17 (18)	2 (13)	1.0
Abnormal chest auscultation	109	44 (40)	34 (36)	10 (67)	0.04
Vital signs at presentation					
Oliguria [b]	109	42 (39)	30 (32)	12 (80)	0.001
Temperature ° Celsius	109	37.1 (36.8-37.6)	37.2 (36.8-37.6)	36.8 (36.4-38.4)	0.39
Supplemental oxygen given	109	23 (21)	13 (14)	10 (67)	<0.001
SpO_2/FiO_2	109	462 (450-471)	462 (457-471)	233 (157-467)	0.001
Respiratory rate	109	20 (18-24)	20 (18-22)	24 (19-33)	0.06
Heart rate	109	99 (80-115)	97 (79-110)	120 (110-143)	<0.001
Systolic blood pressure [c]	109	107 (96-120)	108 (99-120)	101 (91-124)	0.33
Vasopressors	109	42 (39)	28 (30)	14 (93)	<0.001
Impaired consciousness	109	6 (6)	2 (2)	4 (27)	0.003
Disease severity score					
SPiRO score [d]	109	1 (0-2)	1 (0-2)	3 (2-3)	<0.001

Number (percentage) or median (interquartile range) presented

[a] Pulmonary haemorrhage defined as documented frank haemoptysis or frank blood present on tracheal aspirate

[b] Documented urine output of less than 0.5 mL/kg/hour

[c] In the patients in whom vasopressor therapy was not initiated

[d] Three-point SPiRO score (Systolic blood Pressure < 100 mmHg, Respiratory auscultation abnormalities, Oliguria), each is awarded one point [4]

Table S12. Haematological indices of individuals admitted to a referral hospital with leptospirosis in Far North Queensland, January 2015–June 2024, and the association of these factors with the requirement for intubation and mechanical ventilation.

Variable	Reference range [a]	Number with data	All n=109	Not intubated n=94	Intubated n=15	P
Haemoglobin initial	115 - 160 g/dL	109	133 (122-147)	134 (123-149)	123 (114-135)	0.03
Haemoglobin lowest	115 - 160 g/dL	109	112 (98-123)	115 (104-123)	81 (69-97)	<0.0001
White cell initial	4.0 - 11.0 $x10^9$/L	109	9.3 (6.9-12.1)	8.9 (6.2-11.5)	11.6 (9.9-13.7)	0.003
White cell highest	4.0 - 11.0 $x10^9$/L	109	13.1 (9.6-18.4)	12.0 (9.1-15.6)	24.2 (22.1-33.3)	<0.0001
Platelet initial	140 - 400 $x10^9$/L	109	114 (70-168)	130 (78-176)	40 (35-61)	<0.0001
Platelet count lowest	140 - 400 $x10^9$/L	109	82 (31-121)	95 (56-127)	22 (15-26)	<0.0001
Neutrophil initial	2.0 - 8.0 $x10^9$/L	109	8.2 (5.5-10.7)	7.7 (5.3-10.4)	10.4 (8.7-12.7)	0.003
Neutrophil highest	2.0 - 8.0 $x10^9$/L	109	11.1 (8.3-14.6)	10.5 (7.2-12.7)	21.2 (19.9-27.8)	<0.0001
Lymphocyte initial	1.0 - 4.0 $x10^9$/L	109	0.5 (0.3-0.7)	0.5 (0.3-0.7)	0.5 (0.3-0.6)	0.85
Lymphocyte lowest	1.0 - 4.0 $x10^9$/L	109	0.3 (0.2-0.5)	0.3 (0.2-0.5)	0.4 (0.2-0.5)	0.67
INR initial	0.9 - 1.2	83	1.1 (1.1-1.3)	1.1 (1.1-1.3)	1.1 (1.1-1.2)	0.66
INR highest	0.9 - 1.2	83	1.2 (1.1-1.3)	1.2 (1.1-1.3)	1.2 (1.1-1.3)	0.48
APTT initial	25 - 38 seconds	83	31 (28-34)	31 (28-34)	31 (30-34)	0.38
APTT highest	25 - 38 seconds	83	32 (30-36)	32 (30-36)	34 (31-56)	0.08

The median (interquartile range) is presented. INR: International normalised ratio. APTT: Activated partial thromboplastin time

[a] Queensland public hospital laboratory reference ranges.

Table S13. Biochemical indices of individuals admitted to a referral hospital with leptospirosis in Far North Queensland, January 2015–June 2024, and the association of these factors with the requirement for intubation and mechanical ventilation.

Variable	Reference range [a]	Number with data	All n=109	Not intubated n=94	Intubated n=15	P
Initial serum sodium	135- 145 mmol/L	109	133 (129-135)	133 (130-135)	133 (127-136)	0.60
Lowest serum sodium	135- 145 mmol/L	109	132 (129-135)	132 (129-135)	129 (126-134)	0.09
Initial serum potassium	3.5 - 5.2 mmol/L	109	3.7 (3.4-4.0)	3.7 (3.4-4.0)	3.5 (3.0-3.9)	0.28
Highest serum potassium	3.5 - 5.2 mmol/L	109	4.4 (4.0-4.9)	4.3 (4.0-4.8)	5.0 (4.5-5.4)	0.001
eGFR initial	>90 mL/min/1.73 m^2	97	64 (25-90)	69 (29-90)	33 (18-57)	0.007
eGFR lowest	>90 mL/min/1.73 m^2	97	38 (14-78)	43 (17-83)	14 (10-18)	0.002
Initial serum creatinine	45 - 90 µmol/L	109	113 (88-205)	108 (86-190)	232 (109-314)	0.002
Highest serum creatinine	45 - 90 µmol/L	109	179 (102-382)	168 (97-324)	433 (342-524)	0.0006
Initial serum bicarbonate	22 - 32 mmol/L	109	23 (21-25)	24 (22-25)	20 (19-24)	0.009
Lowest serum bicarbonate	22 - 32 mmol/L	109	20 (17-22)	20 (18-22)	18 (15-20)	0.02
Initial serum bilirubin	<20 µmol/L	109	18 (12-28)	18 (11-27)	22 (14-34)	0.08
Highest serum bilirubin	<20 µmol/L	109	26 (19-48)	25 (17-45)	50 (27-94)	0.002
Initial serum ALT	<34 IU/ml	109	68 (27-115)	68 (27-113)	71 (36-115)	0.59
Highest serum ALT	<34 IU/ml	109	121 (68-208)	122 (65-210)	115 (86-190)	0.94
Initial serum AST	<31 IU/ml	109	63 (34-135)	62 (33-134)	80 (44-201)	0.16
Highest serum AST	<31 IU/ml	109	131 (74-210)	122 (66-205)	175 (108-251)	0.11
Initial serum GGT	<38 IU/ml	109	51 (22-120)	51 (22-156)	56 (25-93)	0.86
Highest serum GGT	<38 IU/ml	109	135 (70-235)	137 (68-242)	111 (70-241)	0.75
Initial serum SAP	30 - 110 IU/ml	109	98 (67-171)	97 (66-171)	102 (64-174)	0.80
Highest serum SAP	30 - 110 IU/ml	109	146 (109-208)	148 (107-214)	140 (91-208)	0.81
Initial serum CK	34-145 IU/ml	82	281 (104-1020)	209 (102-757)	965 (347-2270)	0.009
Highest serum CK	34-145 IU/ml	82	350 (114-1020)	215 (104-820)	980 (446-3480)	0.0006
Initial serum CRP	<5 mg/L	105	187 (138-287)	178 (137-272)	274 (168-333)	0.002
Highest serum CRP	<5 mg/L	105	227 (159-323)	221 (158-313)	274 (168-376)	0.0006
Initial serum lactate	0.5 - 2.2 mmol/L	104	1.5 (1.1-2.3)	1.5 (1.1-2.0)	2.5 (1.8-5.0)	0.0001
Highest serum lactate	0.5 - 2.2 mmol/L	104	2.0 (1.4-2.7)	1.9 (1.3-2.4)	5.3 (2.6-7.6)	<0.0001
Elevated initial serum troponin [c]	-	109	25 (23%)	14 (15)	11 (73)	<0.0001
Elevated serum troponin during hospitalisation [c]	-	109	29 (26%)	18 (19)	11 (73)	<0.0001

The median (interquartile range) or the absolute number (percentage) is presented. ALT: alanine aminotransferase; AST: aspartate aminotransferase; GGT: gamma glutamyl transferase; SAP: serum alkaline phosphatase; CK: creatinine kinase; CRP: C-reactive protein.

[a] Queensland public hospital laboratory reference ranges. [b] Troponin is expressed as a categorical variable as the Beckman Coulter assay for troponin I (normal reference range <0.040 µg/L) was replaced by the Siemens Atellica assay (reference range <20 ng/L normal for men; <10 ng/L normal for women) during the study period

Table S14. The presentation, management and clinical course of the six children with leptospirosis in Far North Queensland, January 2015–June 2024.

Age, gender	Comorbidity	How was leptospirosis diagnosed? Inferred serovar if known.	Pulmonary involvement	Pulmonary haemorrhage	Antibiotic therapy	Duration of symptoms prior to antibiotics	Escalation of care	Length of hospitalisation and outcome
13 Male	No	Blood PCR. Serovar Australis	Yes	Yes	Vancomycin, meropenem, ceftriaxone	7 days	Admitted to ICU for vasopressor support, corticosteroids, RRT and mechanical ventilation. Transferred to a quaternary centre for specialist paediatric ICU care.	26 days; survived
13 Male	No	Blood PCR	Yes	Yes	Ceftriaxone, doxycycline, metronidazole	2 days	Admitted to ICU for vasopressor support, corticosteroids and mechanical ventilation	7 days; survived
13 Male	No	Blood PCR	Yes	No	Ceftriaxone, doxycycline, gentamicin	2 days	Admitted to ICU for monitoring	6 days; survived
15 Male	No	Blood PCR	No	No	Ceftriaxone, doxycycline	2 days	No	5 days; survived
11 Male	No	Initial serology. Serovar Zanoni	No	No	No antibiotics	-	No	2 days; survived
7 Male	No	Convalescent serology. Serovar Zanoni	No	No	No antibiotics	-	No	6 days; survived

PCR: polymerase chain reaction; ICU: Intensive care unit
